# Unveiling the Heartbeat of Healing: Exploring Organizational Culture in a Tertiary Hospital’s Emergency Medicine Department and Its Influence on Employee Behavior and Well-Being

**DOI:** 10.3390/ijerph21070912

**Published:** 2024-07-12

**Authors:** Roshni D’Silva, Jayaraj Mymbilly Balakrishnan, Tarushree Bari, Reena Verma, Rajesh Kamath

**Affiliations:** 1 Department of Social and Health Innovation, Prasanna School of Public Health, Manipal Academy of Higher Education, Manipal 576104, Karnataka, India; roshni.psphmpl2022@learner.manipal.edu; 2 Department of Emergency Medicine, Kasturba Medical College, Manipal, Manipal Academy of Higher Education, Manipal 576104, Karnataka, India; jayaraj.mb@manipal.edu; 3 Directorate of Online Education, Manipal Academy of Higher Education, Manipal 576104, Karnataka, India; tarushree.bari@manipal.edu; 4 Welcomgroup Graduate School of Hotel Administration, Manipal Academy of Higher Education, Manipal 576104, Karnataka, India

**Keywords:** emergency medicine department (EMD), organizational culture, employee well-being, burnout, job satisfaction, stress

## Abstract

This study examined the organizational culture of an emergency medicine department (EMD) in a tertiary hospital in Karnataka, India, using a prospective cross-sectional design from January to February 2024. It aimed to identify the predominant and supporting organizational cultures within the EMD and their influence on employee behavior and well-being, including job satisfaction, burnout, stress levels, and coping strategies. A total of 82 participants, including physicians, emergency medical technicians, and nurses, completed the Organizational Culture Assessment Instrument (OCAI) and a self-designed questionnaire. Ethical clearance was obtained (IEC2-656). Clan culture emerged as the dominant culture (73.17%), emphasizing collaboration and adaptability, correlated with lower stress levels and high job satisfaction (90.78%). Emotional exhaustion was the most common burnout symptom (53.66%). The coping strategies varied, with employees in Clan cultures seeking social support, while those in Hierarchy cultures sought guidance from superiors. This study highlighted the significant role of organization culture in employee well-being and EMD effectiveness, influenced by social values like respect for authority. The limitations included single-setting analysis, an uneven subgroup representation, and a lack of qualitative insights. Future research should involve multiple hospitals and qualitative methods for a comprehensive understanding.

## 1. Introduction

An emergency medicine department (EMD) operates in a uniquely high-pressure environment compared to other hospital departments [1,2]. It handles unpredictable patient volumes, provides immediate acute care, and requires rapid clinical judgments in critical situations [1,2,3]. There is always a risk that EMD staff may face aggression or violence from the public [1,2]. Overcrowding in an EMD is also a widespread phenomenon that causes delay in treatment, leading to increased mortality and morbidity rates, further contributing to stressful events between patients and EMD staff [4]. This dynamic and unpredictable nature makes the EMD distinct from other departments that often deal with more predictable types of patients. For instance, the orthopedic department often deals with predictable types of patients, such as those with sports injuries or fractures, while, in neurosurgery, patients commonly present with conditions like traumatic brain injuries or strokes. In contrast, the EMD faces a significantly broader spectrum of patient presentations. Patients arriving at the EMD can have diverse medical emergencies, ranging from acute myocardial infarctions and respiratory distress to severe trauma and poisoning [5]. In addition, studying organizational culture in the EMD is similar to examining the culture in a “mini-hospital”. It provides a comprehensive range of patient care and administrative processes within a condensed timeframe. Every patient in the EMD undergoes registration, diagnostic tests, therapeutic interventions, and a discharge process [6]. This diverse environment, which prioritizes versatility, rapid adaptation, and interdisciplinary collaboration among healthcare teams, necessitates studying the prevalent organizational culture to understand its impacts on employee behavior and well-being.

Emergency and injury cases annually constitute between 9% and 13% of all patients presenting to health facilities [7]. On any given day, emergency cases account for 11% to 30% of all outpatient department (OPD) patients [7]. These cases translate to 19% to 24% of admissions in government hospitals and 31% to 39% of admissions in private hospitals [7,8]. Road traffic injuries (RTIs) are a significant global public health issue. Annually, RTIs result in approximately 1.35 million deaths and over 50 million injuries worldwide [9]. In India, the burden of medical emergencies, including road traffic injuries (RTIs) and heart attacks is very high. In 2021, 1.5 lakh people lost their lives in road accidents, averaging 18 deaths per hour [10]. These statistics highlight the critical role of EMDs in the healthcare system.

The importance of organizational culture has gained more attention over the past two decades, as it has served as a key explanatory variable behind workplace behavior and performance [11,12,13]. Organizational culture represents the shared elements of institutional life within a workplace or organization. These elements include common beliefs, assumptions, attitudes, activities, behaviors, practices, and interactions among members. Over time, these shared ways of thinking and behaving become norms, reflecting what is considered legitimate and acceptable in that specific workplace or organization. Based on these ideas, we can define culture as the collective combination of shared attributes, values, thought processes, and behaviors exhibited by individuals within workplaces or organizations [14,15].

The organizational culture of any institution impacts employee well-being and mental health [16]. A supportive work culture that prioritizes employee happiness and job satisfaction tends to yield higher levels of productivity by driving its employees’ conduct in a certain direction in order to accomplish organizations’ goals. This improves employees’ performance and increases the feeling of job satisfaction, leading to improved employee retention and organizational performance [16,17]. Additionally, these supportive environments offer opportunities for professional growth, recognition, and work–life balance, all of which can enhance employees’ overall well-being. Conversely, unfavorable organizational cultures that foster stress, conflict, and toxicity can have numerous detrimental effects on both employees and organizations. Such cultures negatively impact employee well-being, leading to high levels of stress, feelings of being unsupported or undervalued, and difficulties in balancing work and personal life. This can result in increased absenteeism, decreased job satisfaction, and reduced productivity. Moreover, negative work cultures can lead to higher turnover rates and lower levels of employee engagement, ultimately affecting organizational success and reputation [16]. These issues can be exacerbated in a high-demand and stressful department like the EMD. Healthcare organizations prioritize organizational culture because the shared beliefs, values, and feelings within the organization or department guide perceptions and approaches to the work to be accomplished. If aspects of the organizational culture are ill-defined, frequently shifting, poorly communicated, not reinforced, and poorly supported administratively, employees’ collective perceptions and behaviors (e.g., delivery of care, safe work practices, and teamwork) will be inconsistent [18].

Prominent works in the field of organizational culture include “Hofstede’s model” by Hofstede, the “Organizational Culture Profile (OCP) model” by O’Reilly et al., the “Organizational Culture Assessment Instrument (OCAI)” by Cameron and Quinn, the “Revised Organizational Culture Profile (ROCP) model” by Sarros et al., and the “Model of Organizational Culture and Effectiveness” by Denison [19]. For this paper, the Organizational Culture Assessment Instrument (OCAI) was used. The fundamentals of the OCAI are derived from the “Competing Values Framework” (CVF), conceptualized and articulated by Cameron and Quinn [20,21]. This model was selected based on research findings by Heritage et al., which confirmed the psychometric properties of the OCAI, validating its effectiveness as a reliable instrument for assessing organizational culture [22]. Another reason for selecting the Competing Values Framework over other models is its proven effectiveness in diagnosing the dominant organizational culture in hospitals [23,24]. In this study, CVF is used to assess and diagnose the existing culture within the EMD of the tertiary hospital under study. By categorizing organizational culture into four types—Clan, Adhocracy, Market, and Hierarchy—the framework helps identify dominant cultural characteristics and underlying values.

**Figure 1:**
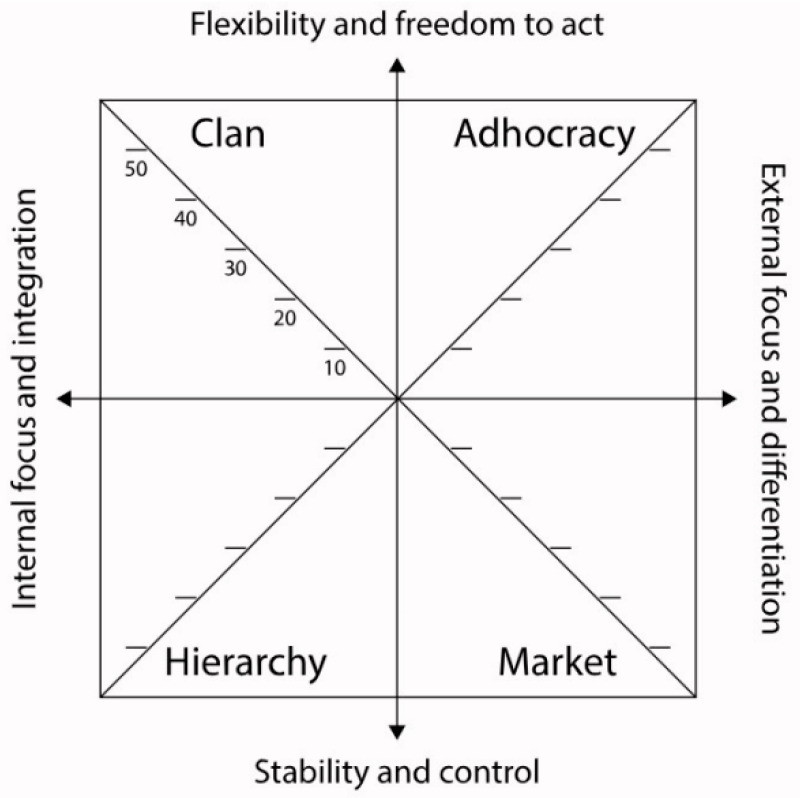
Cameron and Quinn’s Competing Values Framework (CVF)—mapping organizational culture types. Source: https://www.ocai-online.com/about-the-Organizational-Culture-Assessment-Instrument-OCAI (accessed on 2 July 2024) [25].

Based on two critical variables, internal–external orientation and stability and the flexibility dimension, the CVF creates a four-quadrant model. The vertical axis is characterized by flexibility and control, while the horizontal axis has internal and external focus as its endpoints. These two axes delineate four distinct cultural types: Clan culture, Adhocracy culture, Market culture, and Hierarchy culture, as shown in Figure 1 [25]. When applied to the context of emergency medicine, this framework offers a systematically organized viewpoint for understanding the prevailing organizational culture.

Clan culture prioritizes employee well-being, cohesiveness, engagement, and teamwork through human affiliation, collaboration, trust, loyalty, and support. Managers in this culture work collectively to inspire and motivate their employees, cultivating a culture of excellence and commitment which encourages teamwork and empowerment of healthcare professionals to ensure effective patient care. Adhocracy culture fosters innovation, creativity, and adaptability, encouraging a dynamic and inventive approach to healthcare delivery. Managers allocate resources for research and development, inspiring healthcare professionals to pursue innovative approaches in medical practice. Hierarchy culture is characterized by strict rules and regulations governing organizational activities to ensure efficient and controlled management. It involves establishing effective control systems to ensure adherence to medical procedures and regulations. Clear communication, consistency, and stability are valued to ensure efficiency and effectiveness in emergency healthcare provision. Market culture focuses on addressing competition and market achievement to meet the department’s goals and objectives. It entails gathering patient and competitor information, setting appropriate goals and adopting task-focused leadership. Managers in this culture concentrate on the effectiveness of external factors, ensuring competitiveness and achieving high-quality patient care [26]. Each of these cultures possesses distinct strengths and challenges. Identifying the prevailing culture in an EMD allows hospital administrators to capitalize on its strengths and strategically address its weaknesses.

The World Health Organization (WHO) defines mental health as “a state of mental well-being that enables people to cope with the stresses of life, realize their abilities, learn well and work well and contribute to their community” [27]. This definition highlights the multifaceted nature of mental well-being and its centrality to individuals’ ability to function effectively in various spheres of life. Workplace conditions determine the extent to which employees perceive that their workplaces help them meet the criteria outlined in the above definition. Working in the EMD presents stressful events such as patient deaths or participation in resuscitation efforts. These can impact its staff members, presenting emotional and physical challenges. Despite the frequent exposure to such stressful events, EMD staff do not develop immunity to the stress they induce. Moreover, they are often insufficiently prepared and lack adequate support to cope with these challenges. Therefore, management should prioritize investing in employee well-being practices [28,29]. Employee well-being is defined as the overall health of employees, including their mental, physical, emotional, and financial well-being [30]. Without proper support, workplace stress can have harmful effects on the physical, psychological, and emotional well-being of EMD staff, leading to symptoms of burnout [28]. Burnout is defined as “a state of vital exhaustion” in the International Classification of Diseases (ICD-11) and is considered the most useful measure of barriers to professional well-being [31]. It is a syndrome marked by emotional exhaustion, depersonalization, and a diminished sense of personal achievement [32].

According to the recent literature, EMD physicians exhibit significantly higher burnout rates compared to their counterparts in other medical specialties [33]. Research indicates that the prevalence of burnout among EMD physicians can reach values as high as 60% in some studies. In contrast, EMD nurses, as assessed by the Maslach Burnout Inventory (MBI), demonstrate a lower, yet still concerning, prevalence of burnout, with approximately 30% of them meeting at least one burnout criterion [31]. Hence, understanding which organizational culture among Clan, Adhocracy, Market, and Hierarchy may contribute to employee burnout is crucial, as it will allow healthcare institutions, especially those in the EMD, to tailor their work environments and management strategies.

Constant exposure to critical cases, long working hours, and high patient volumes results in reduced job satisfaction. Job satisfaction is a sentiment that reflects the cognitive and behavioral responses of employees towards their job [34]. Job satisfaction, as a broad concept, refers to an employee’s overall feelings toward their job, encompassing specific aspects such as supervision, compensation, opportunities for advancement, and building morale [35]. Reduced job satisfaction is linked to higher staff turnover rates, further straining EMD resources. The existing literature suggests a direct link between organizational culture, job satisfaction, and turnover intention. Organizational culture significantly impacts an employee’s intention to leave [35,36]. Recognizing the predominant culture within a hospital and actively working to modify it can play a pivotal role in motivating and retaining employees.

The WHO predicts a shortage of 10 million healthcare workers by 2030, predominantly in low- and lower-middle-income countries. Countries across different socioeconomic levels encounter challenges in educating, hiring, placing, retaining, and enhancing the performance of their workforce to varying extents [37]. In a field where experienced professionals are invaluable, retaining skilled staff ensures continuity of care and promotes a sense of stability within the department. Prioritizing staff well-being through mental health support, adequate rest and work–life balance initiatives becomes crucial in preventing and mitigating burnout.

This paper explores the organizational culture within the EMD of a tertiary hospital in Karnataka, India. There is a lack of comprehensive studies examining the specific organizational culture within EMDs and how it affects employees. Understanding these cultural dynamics is crucial due to the high-stress environment and critical nature of EMDs, which significantly impact patient outcomes, staff well-being, and the overall departmental effectiveness. Identifying the prevailing culture can inform targeted interventions to enhance job satisfaction, reduce burnout, and improve the quality of patient care, particularly in culturally diverse settings like Indian hospitals. It will also help in fostering a supportive and effective workplace. This study aims to identify the predominant organizational culture, along with any supporting cultures, within an emergency medicine department and explore its influence on employee behavior. Additionally, it seeks to analyze the well-being of employees, encompassing job satisfaction, burnout, stress levels, and the respective coping strategies in relation to the perceived organizational culture. Coping strategies are cognitive, behavioral, or emotional methods that individuals develop to handle stressful situations and preserve their mental and physical well-being. Essentially, they are techniques used to manage stress and reduce the impact of stressors, thereby enhancing physical and emotional health [38]. Ultimately, this study intends to provide recommendations for enhancing employee well-being and overall performance through potential improvements in the organizational culture of the emergency medicine department. This study intends to have an exploratory approach to unravel the complex relationship between organizational culture, employee behavior, and well-being in the dynamic environment of emergency medicine and form a basis for future research.

We have formulated the following hypotheses for our study:

1. There will be no significant difference in cultural preferences among the employees in the EMD (H_1_).
2. There is an association between organizational culture and profession (H_2_).
3. Employees perceiving a Clan culture will report higher job satisfaction and lower levels of burnout and stress compared to those in other cultural environments (H_3_).

The relevance of this research is underscored by its contribution to the existing body of knowledge on organizational behavior and healthcare management. While a previous study by Nambiyar P. explored organizational culture in healthcare settings, there exists a gap in understanding its nuances within the context of EMDs in Indian tertiary care hospitals, which distinguishes our research from the former [39]. By bridging this gap, this research will provide valuable insights into tailoring management practices, enhancing employee morale, and, ultimately, improving patient care quality.

## 2. Methodology

### 2.1. Study Design and Setting

The study utilized a prospective cross-sectional research design to capture a snapshot of the perceived organizational culture within the emergency medicine department (EMD) of a tertiary hospital [40,41]. This approach facilitated an immediate understanding of the current state of the organizational culture, employee behavior, and well-being without requiring long-term follow-up. The immediacy provided by the cross-sectional design enabled timely decision making and intervention planning based on the current findings, enhancing the effectiveness of organizational management within the EMD. This study was conducted within the EMD of a tertiary hospital in Karnataka, India.

### 2.2. Study Duration

This study extended over a two-month period, spanning from January 2024 to February 2024.

### 2.3. Methods and Measurements

#### 2.3.1. Sample Size and Selection

The EMD comprised 17 physicians, 8 emergency medical technicians (EMTs), and 106 nurses. To adjust for potential dropouts, the formula proposed by Kothari was employed, resulting in a calculated sample size of 82 participants, including 8 physicians, 8 EMTs, and 66 nurses [42,43].

This sample size was determined using a 5% margin of error (A), with a Z-score (Z) of 1.96 corresponding to the 95% confidence level, an expected response rate (R) of 80%, and a population proportion (P) of 50%.

$$n=\frac{P\left(1-P\right)}{\frac{A^{2}}{Z^{2}}+\frac{P\left(1-P\right)}{NR}}$$

The study participants consisted of physicians, nurses, and emergency medical technicians who voluntarily agreed to participate. Administrative and support staff, as well as those who did not voluntarily agree to participate, were excluded from this study.

#### 2.3.2. Data Collection

Formal invitations were extended to physicians, nurses, and EMTs within the EMD, and comprehensive information about this study’s purpose was provided to them. Written informed consent was obtained from the participants who voluntarily agreed to take part in this study.

To identify the prevailing organizational culture within the EMD, an Organizational Culture Assessment Instrument (OCAI) was administered to each participant. Next, a self-designed and validated questionnaire was administered to evaluate employee behavior and employee well-being based on the perceived organizational culture. Employee behavior was assessed across various dimensions, including leadership style, communication, teamwork, and other values. The questionnaire also delved into job satisfaction, burnout, stress, and coping strategies to gain insights into employee well-being. The participants were asked to identify sources of stress and the coping mechanisms employed by them to maintain their well-being.

#### 2.3.3. Tools and Materials

The OCAI was utilized to identify the prevailing cultures in the EMD. It is a validated tool for assessing organizational culture, developed by Robert Quinn and Kim Cameron. It is based on the CVF. The assessment of employee behavior and well-being was conducted using a questionnaire designed by a researcher and validated by experts. Both assessment tools used a 5-point Likert Scale to evaluate each component. The constructs for evaluating employee behavior across various cultures were derived from the existing literature on Clan culture, Adhocracy culture, Market culture, and Hierarchy culture. In the evaluation of employee well-being, three components—job Satisfaction, burnout, and stress—were considered. The R programming software was used for statistical analysis.

### 2.4. Statistical Analysis

Data were entered in MS Excel (version 2023) and analyzed using the R programming software (version 4.3.0). Key variables related to organizational culture, employee behavior, and well-being were summarized using descriptive statistics. The Kruskal–Wallis rank sum test was used to evaluate whether there were significant differences in culture preferences among the employees in the EMD [44,45]. A Dunn test was conducted to examine pairwise significant differences in culture preferences among the EMD employees to determine whether specific pairs of culture types significantly differed from one another in terms of preference. The chi-square test was employed to assess the association between organizational culture and employees’ profession (physicians, nurses, EMTs).

### 2.5. Ethical Considerations

Emphasis was placed on maintaining participant confidentiality and anonymity to mitigate biases arising from fear of repercussions. To mitigate stress during the data collection process, the participants were reassured that their contributions were intended to improve the workplace.

Ethical clearance was obtained from the Institutional Ethics Committee (IEC) of the tertiary hospital (IEC2-656) to ensure adherence to ethical guidelines and the protection of participant rights.

## 3. Results

This study involved 82 employees from the EMD of a tertiary care hospital in the Karnataka state of India. The study participants comprised a diverse cohort of clinical professionals including 8 physicians, 66 nurses, and 8 EMTs.

The strength of an organization’s culture is determined by the number of points awarded to a particular culture type. In our study, the highest points were awarded to Clan culture (30.81 points), making it the dominant culture. This was followed by Market culture (23.41 points), Adhocracy culture (23.15 points), and Hierarchy culture (22.62 points), as shown in Table 1.

Table 1 shows the culture profile of the EMD. The culture profile is a mix of the four culture types in the Competing Values Framework (CVF). In our study, the EMD’s culture profile emphasized collaboration, teamwork, mentorship, and employee development.

**Figure 2:**
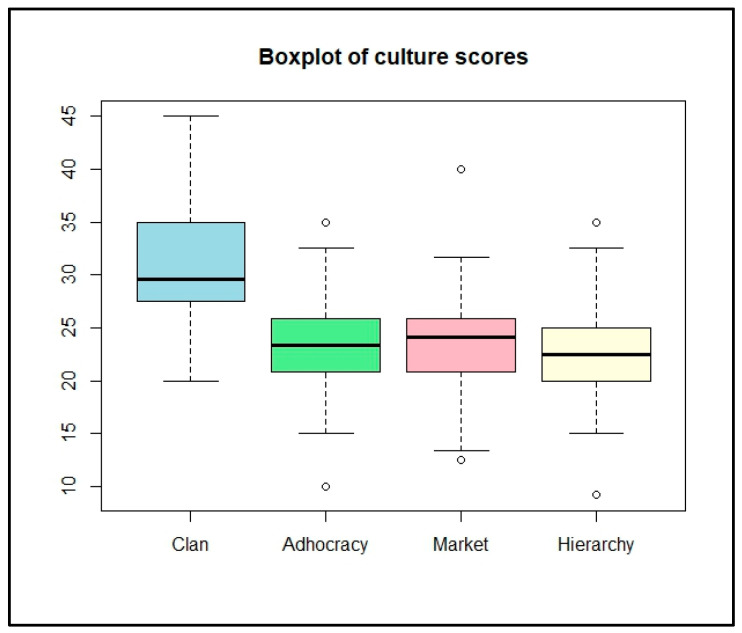
EMD culture profile boxplot.

The boxplot (Figure 2) visually represents the distribution of the culture profile scores across four dimensions: Clan, Market, Adhocracy, and Hierarchy. Each dimension’s score is depicted as a box.

The Clan culture has a median score of 30.81, the Market culture has a median score of 23.41, the Adhocracy culture has a median score of 23.15, and the Hierarchy culture has a median score of 22.62. The box lengths are not uniform. The Clan culture box has a longer length, indicating that the Clan culture has a broader range of scores and, thus, is more prominent in shaping the organizational culture in the EMD. Conversely, cultures with shorter boxes (Adhocracy, Market, and Hierarchy) represent aspects with more consistent scores and less variability, suggesting that they are less prominent in shaping the organizational culture in the EMD.

This study hypothesized that there would be no significant difference in cultural preferences among the EMD employees (H0). A Kruskal–Wallis rank sum test revealed a statistically significant difference (*p* < 0.05), leading to the rejection of H_1_. A subsequent Dunn test showed significant pairwise differences among the Adhocracy–Clan, Adhocracy–Hierarchy, Clan–Hierarchy, Adhocracy–Market, Clan–Market, and Hierarchy–Market culture pairs (*p* < 0.005). These results confirmed a diverse cultural mix in the EMD.

Table 2 presents an overview of the organizational culture distribution within the EMD, as perceived by the employees. The accompanying percentages represent the proportion relative to the total.

This study revealed Clan culture as the dominant culture, with 73.17% of the participants (*n* = 60) perceiving it as the prevalent organizational culture within the EMD. This findings align with the highest culture scores attributed to the Clan culture, as depicted in Table 1, reinforcing the dominance of Clan culture within the EMD.

Table 3 provides a breakdown of the distribution of organizational culture based on the employees’ professions. The accompanying percentages represent the proportion relative to the total.

A chi-square test was conducted to check the association between culture and profession. The results did not yield a significant *p*-value, implying that profession does not influence cultural preferences in the EMD.

### 3.1. Employee Behavior Assessment

To identify the employee behavior associated with a specific organizational culture type, an assessment was designed that included 20 behaviors that collectively encompassed the characteristics of all four culture types. The participants were requested to rate each behavior according to the extent to which it was followed within the EMD using a 5-point Likert scale, where 1 signified “Strongly Disagree” and 5 corresponded to “Strongly Agree”.

In our study, it was found that the employees displayed consistent behaviors in the EMD, regardless of their perceived organizational culture. One possible explanation for this could be that the EMD has a strong organizational identity. Hence, its core values influence employee behavior regardless of the organizational culture. Table 4 illustrates these behaviors.

### 3.2. Employee Well-Being Assessment

Employee well-being was evaluated in three key domains: job satisfaction, burnout, and perceived stress levels.

#### 3.2.1. Job Satisfaction

Table 5 presents the count and respective percentage of agreement among the employees (physicians, nurses, and EMTs) regarding key job satisfaction elements, categorized by the prevailing organizational culture type.

Overall, 98.78% (*n* = 81) of the employees found their work in the EMD to be both meaningful and fulfilling, 93.90% (*n* = 77) of the employees felt valued and recognized for their contributions, 91.46% (*n* = 75) of the employees expressed satisfaction with the work environment and culture in the EMD, 91.46% (*n* = 75) of the employees believed that their work in the EMD aligned with their personal values and goals, and 87.80% (*n* = 72) indicated that they were overall satisfied with their job in the EMD.

#### 3.2.2. Burnout

Burnout was assessed by evaluating the frequency of experiencing the burnout symptoms on a scale of 1 to 5, with 1 being “Never” and 5 being “Very Often”. The burnout symptoms included emotional exhaustion, depersonalization, and reduced personal accomplishment.

Table 6 shows the frequency of experiencing emotional exhaustion among the employees based on each employee’s perception of the organizational culture.

Table 7 shows the frequency of experiencing the feeling of depersonalization among the employees based on each employee’s perception of the organizational culture.

Table 8 shows the frequency of experiencing the feeling of reduced personal accomplishment among the employees based on each employee’s perception of the organizational culture.

Table 9 shows the overall frequency of experiencing emotional exhaustion, depersonalization, and reduced personal accomplishment among the employees in the EMD.

#### 3.2.3. Stress

The assessment of stress involved employees rating their stress levels on a scale from 1 to 5, with 1 representing “Very Low Stress” and 5 representing “Very High Stress”.

Table 10 displays the levels of stress experienced by the employees based on their perceptions of the organizational culture.

**Figure 3:**
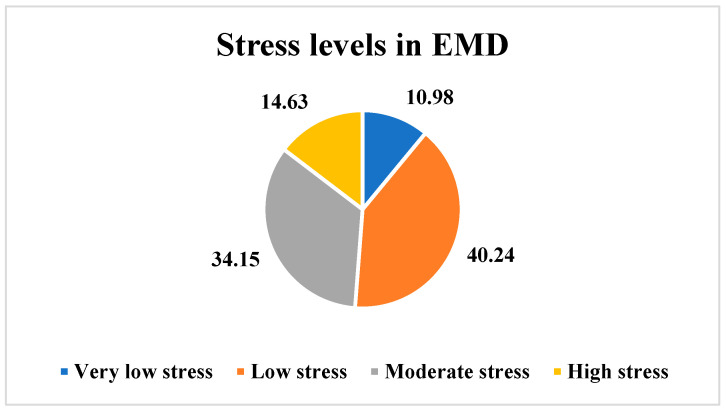
Overall stress levels in the EMD.

Overall, 10.98% of the employees (*n* = 9) perceived very low stress levels, 40.24% of the employees (*n* = 33) perceived low stress levels, 34.15% of the employees (*n* = 28) perceived moderate stress levels, and 14.63% of the employees (*n* = 12) perceived high stress levels in the EMD, as shown in Figure 3.

**Figure 4:**
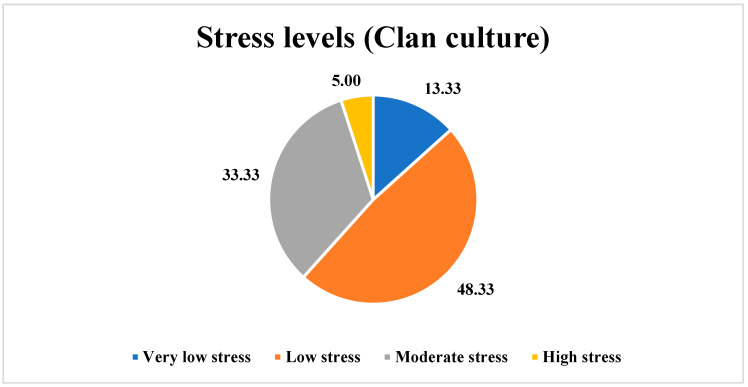
Stress levels among the employees perceiving a Clan culture.

In our study, it was found that, among the employees perceiving a Clan culture, 13.33% (*n* = 8) reported very low stress levels, 48.33% (*n* = 29) reported low stress levels, 33.33% (*n* = 20) reported moderate stress levels, and 5% (*n* = 3) reported high stress levels, as shown in Figure 4.

**Figure 5:**
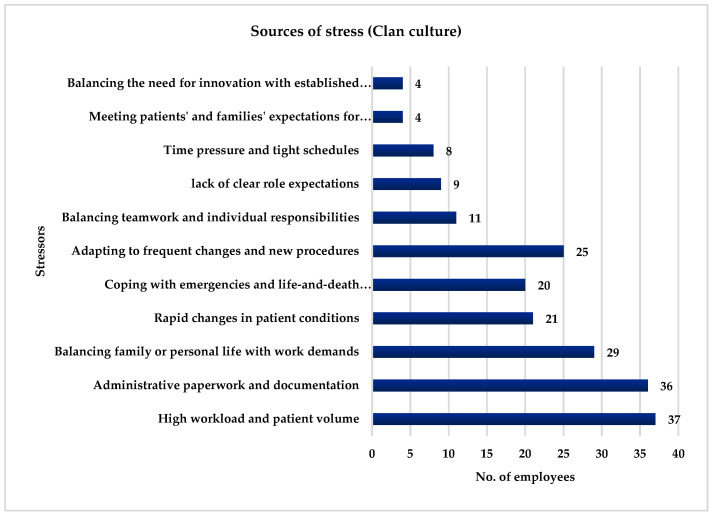
Stressors identified by the employees in a Clan culture.

Figure 5 illustrates the various stressors identified by the surveyed employees in a Clan culture. Among the surveyed employees, 61.67% (*n* = 37) identified high workload and patient volume as significant stressors, while 60% (*n* = 36) highlighted the burden of administrative paperwork and documentation. Balancing family or personal life with work demands was chosen by 48.33% (*n* = 29) of the employees, and 35% (*n* = 21) identified stress arising from rapid changes in patient conditions. Coping with emergencies and life-and-death situations was selected by 33.33% (*n* = 20) of the employees, and 25% (*n* = 15) chose adapting to frequent changes and new procedures. Balancing teamwork and individual responsibilities were a stress factor for 18.33% (*n* = 11) of the employees, while 15% (*n* = 9) pointed to the lack of clear role expectations. Time pressure and tight schedules were identified by 13.33% (*n* = 8) of the employees, and 6.67% (*n* = 4) mentioned the stress of meeting patients’ and families’ expectations for personalized care and the challenge of balancing the need for innovation with established practices as a source of stress.

**Figure 6:**
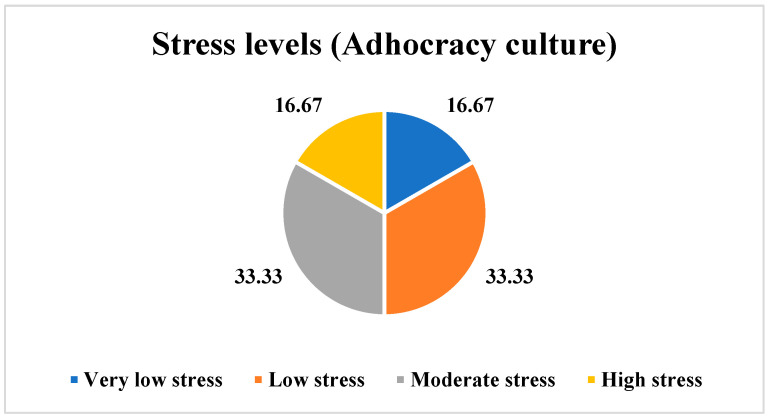
Stress levels among the employees perceiving an Adhocracy culture.

In the case of the employees perceiving an Adhocracy culture, this study revealed that 16.67% of the employees (*n* = 1) reported very low stress, 33.33% of the employees (*n* = 2) reported low stress, another 33.33% of the employees (*n* = 2) reported moderate stress, and 16.67% of the employees (*n* = 1) expressed high stress levels, as shown in Figure 6.

**Figure 7:**
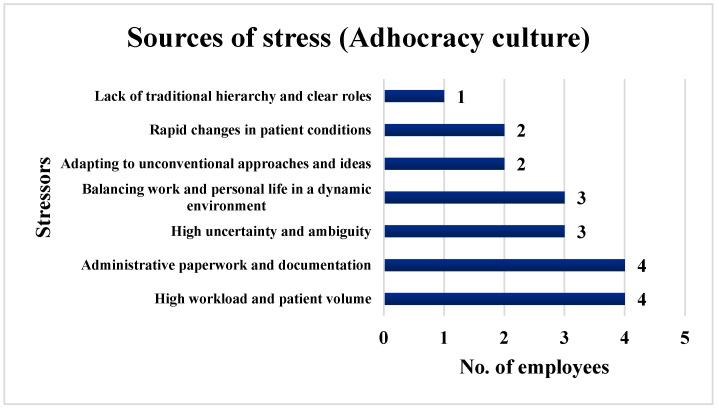
Stressors identified by the employees in an Adhocracy culture.

Additionally, 66.67% of the employees (*n* = 4) selected high workload and patient volume and administrative paperwork and documentation as major sources of stress. Moreover, 50% of the employees (*n* = 3) chose high uncertainty and ambiguity and balancing work and personal life in a dynamic environment, while 33.33% of the employees (*n* = 2) chose adapting to unconventional approaches and ideas and rapid changes in patient conditions as sources of stress. A total of 16.67% of the employees (*n* = 1) pointed to a lack of traditional hierarchy and clear roles as stress-inducing factors. Figure 7 illustrates the stressors identified by the surveyed employees in an Adhocracy culture.

**Figure 8:**
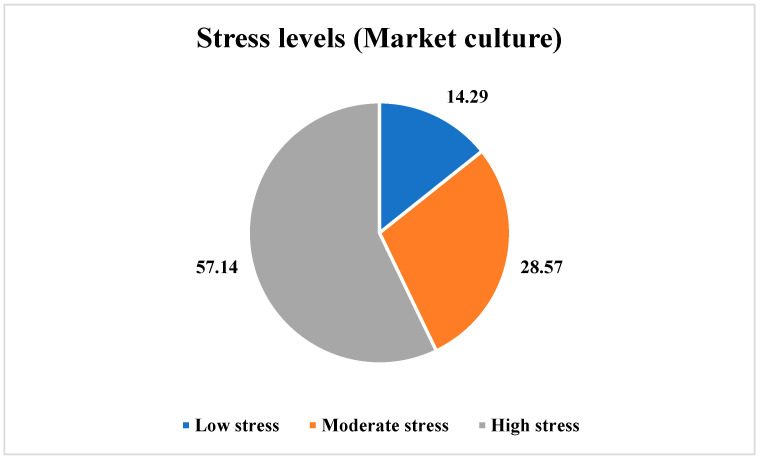
Stress levels among the employees perceiving a Market culture.

In the case of the employees perceiving a Market culture, the findings indicated that 14.29% of the employees (*n* = 1) reported low stress levels, 28.57% of the employees (*n* = 2) reported moderate stress levels, and 57.14% of the employees (*n* = 4) reported high stress levels, as shown in Figure 8.

**Figure 9:**
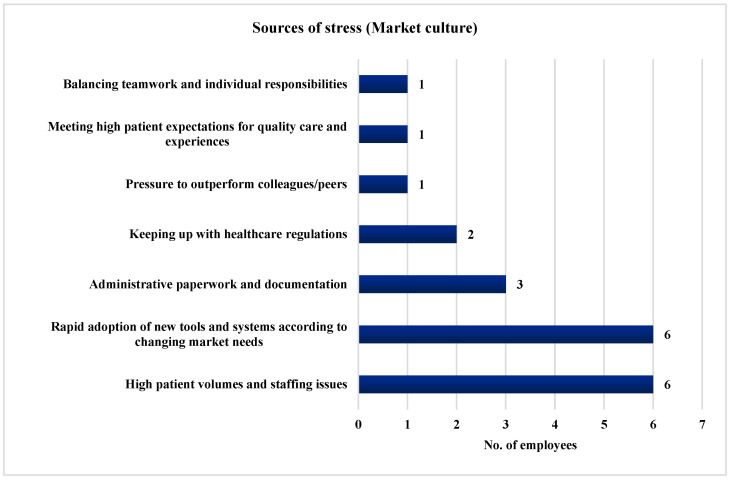
Stressors identified by the employees in a Market culture.

Furthermore, 85.71% of the employees (*n* = 6) identified challenges such as high patient volumes and staffing issues and the swift adoption of new tools and systems to meet changing market demands as significant stressors. Administrative paperwork and documentation were highlighted by 42.86% of the employees (*n* = 3), while 28.57% (*n* = 2) pointed to the stress of keeping up with healthcare regulations. Additionally, 14.29% of the employees (*n* = 1) reported the pressure to outperform colleagues/peers, meeting high patient expectations for quality care and experiences, and the task to maintain the delicate balance between teamwork and individual responsibilities as sources of stress. Figure 9 illustrates the stressors identified by the employees in a Market culture.

**Figure 10:**
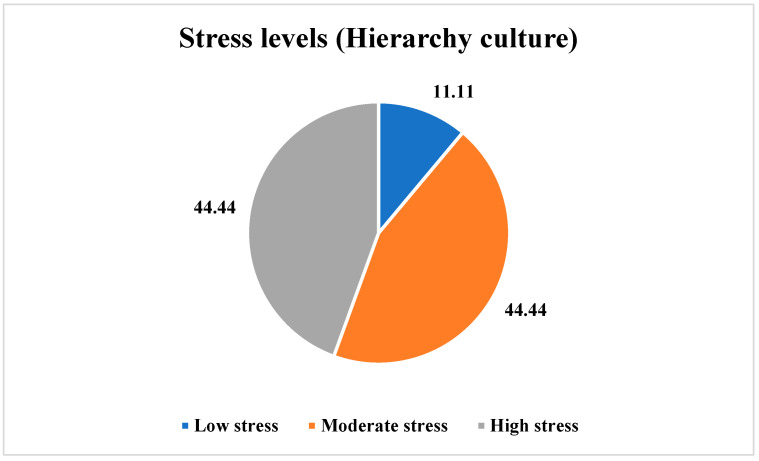
Stress levels among the employees perceiving a Hierarchy culture.

Finally, among the employees perceiving a Hierarchy culture, this study found that 11.11% of the employees (*n* = 1) experienced low stress levels, 44.44% of the employees (*n* = 4) experienced moderate stress levels, and 44.44% of the employees (*n* = 4) experienced high stress levels, as shown in Figure 10.

**Figure 11:**
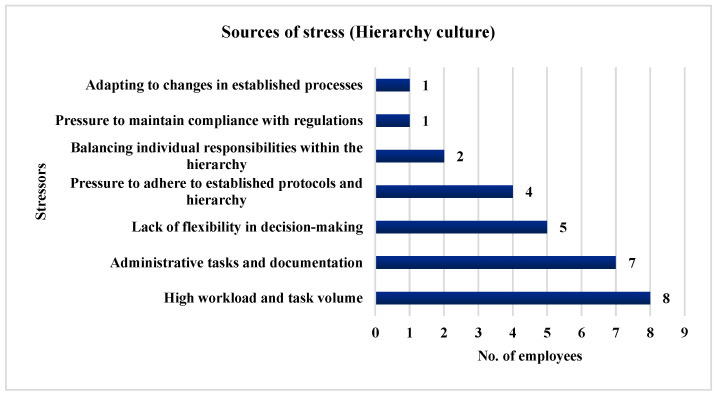
Stressors identified by the employees in a Hierarchy culture.

Additionally, 88.89% of the employees (*n* = 8) highlighted high workload and task volume as major stressors, while 77.78% of the employees (*n* = 7) identified administrative tasks and documentation as significant contributors to stress. A lack of flexibility in decision making was chosen by 55.56% of the employees (*n* = 5), and 44.44% (*n* = 4) reported the pressure to adhere to established protocols and hierarchy as stress factors. Furthermore, 22.22% of the employees (*n* = 2) pointed to balancing individual responsibilities within the hierarchy as a source of stress, and 11.11% of the employees (*n* = 1) highlighted the pressure to maintain compliance with regulations and adapt to changes in established processes as additional sources of stress. Figure 11 illustrates the stressors identified by the employees in a Hierarchy culture.

**Figure 12:**
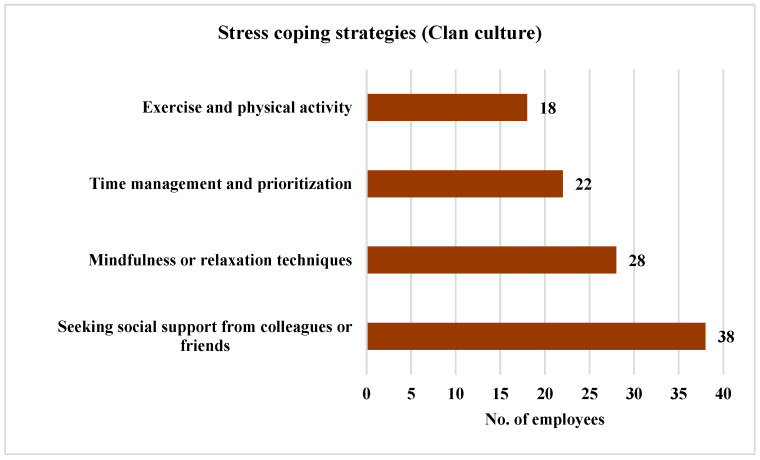
Strategies employed by the employees perceiving a Clan culture to cope with stress.

The strategies employed by the employees who perceived a Clan culture to cope with stress and enhance their well-being within the EMD were investigated. The findings are shown in Figure 12. The results indicated that 63.33% of these employees (*n* = 38) favored seeking social support from colleagues or friends to reduce stress. Then, 46.67% of the employees (*n* = 28) opted for mindfulness or relaxation techniques, while 36.67% of employees (*n* = 22) managed their stress through time management and prioritization. A total of 30% of the employees (*n* = 18) opted for exercise or engaging in physical activity as a stress-reduction method.

**Figure 13:**
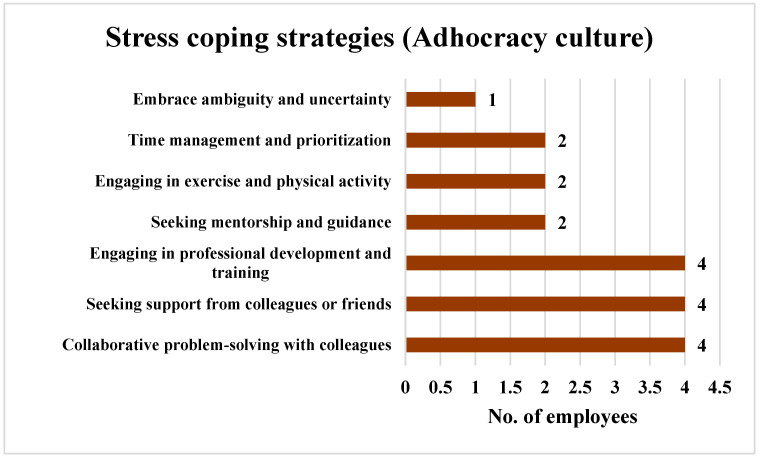
Strategies employed by the employees perceiving an Adhocracy culture to cope with stress.

Similarly, when the employees perceiving an Adhocracy culture were queried about the strategies employed to cope with stress and enhance the well-being within the EMD, the findings revealed that 66.67% of them (*n* = 4) opted for collaborative problem solving with colleagues, seeking support from colleagues or friends, and engaging in professional development and training as methods to reduce stress. Another 33.33% of the employees (*n* = 2) managed their stress through seeking mentorship and guidance, engaging in exercise and physical activity, and time management and prioritization. Additionally, 16.67% of the employees (*n* = 1) chose to embrace ambiguity and uncertainty to manage their stress. The findings are shown in Figure 13.

**Figure 14:**
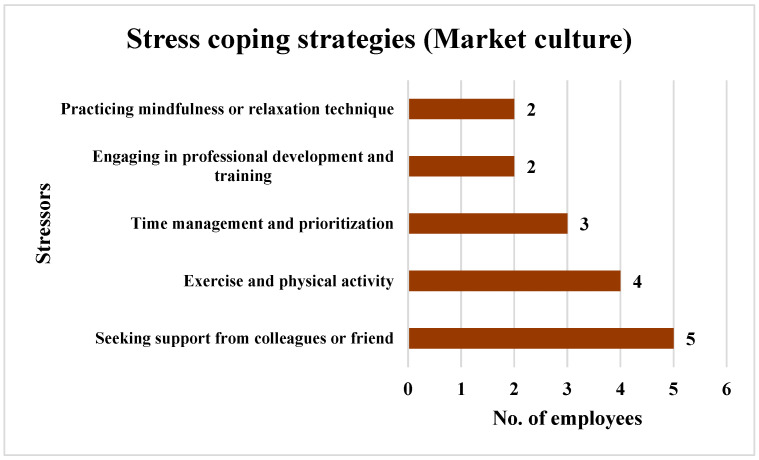
Strategies employed by the employees perceiving a Market culture to cope with stress.

In the case of the employees who perceived a Market culture, the results indicated that 71.43% of these employees (*n* = 5) favored seeking support from colleagues or friends to reduce stress. Exercise and physical activity were chosen by 57.14% of the employees (*n* = 4), and 42.86% of the employees (*n* = 3) managed their stress through time management and prioritization. A total of 28.57% of the employees (*n* = 2) opted to engage in professional development and training and practice mindfulness or relaxation techniques to manage their stress. The findings are shown in Figure 14.

**Figure 15:**
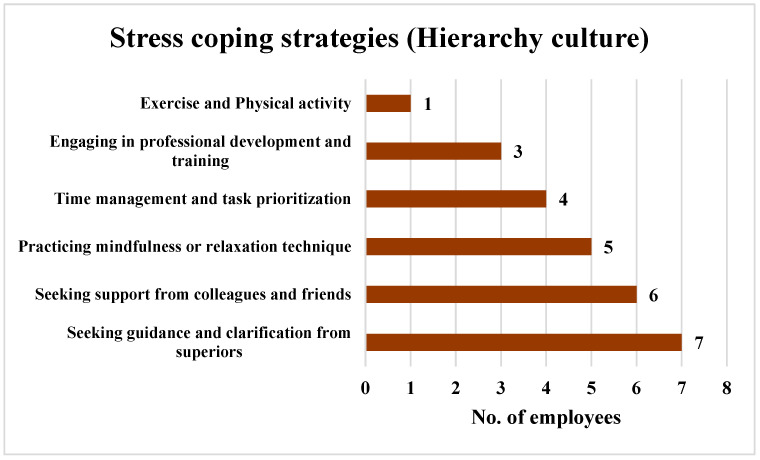
Strategies employed by the employees perceiving a Hierarchy culture to cope with stress.

In the case of the employees who perceived a Hierarchy culture, the results indicated that 77.78% of these employees (*n* = 7) favored seeking guidance and clarification from superiors as a method to reduce stress. A total of 66.67% of the employees (*n* = 6) chose to seek support from colleagues and friends, while 55.56% of the employees (*n* = 5) managed their stress by practicing mindfulness or relaxation techniques. A total of 44.44% of the employees (*n* = 4) opted for time management and task prioritization, 33.33% of the employees (*n* = 3) chose to engage in professional development and training, and 11.11% of the employees (*n* = 1) chose to exercise and engage in physical activity to manage their stress. The findings are shown in Figure 15.

## 4. Discussion

This study provided insightful findings regarding the organizational culture within the EMD of a tertiary care hospital in the Karnataka state of India and its influence on employee behavior and well-being.

### 4.1. Organizational Culture Profile

The EMD exhibited a dominant Clan culture, emphasizing collaboration, teamwork, mentorship, and employee development [46]. This was evidenced by the Clan culture receiving the highest score of 30.81 points in the culture assessment and 73.17% of employees perceiving the Clan culture as the most prevalent culture in the EMD. The Clan culture stemmed from the EMD’s focus on providing patient-centric care through coordinated team efforts. Additionally, the Market, Adhocracy, and Hierarchy cultures had moderate representation, indicating that the EMD also valued achievement, innovation, and structure to an extent. These study findings align with the findings of Mannion R et al., who stressed that hospitals are not just one culture but a mix of many, each affecting how people behave and feel [47,48]. This balanced culture profile allowed the EMD to reap the benefits of all four culture types based on situational needs. For instance, onboarding new team members may require extensive shadowing and mentoring, aligning with the Clan culture principles, while responding to new disease outbreaks may call for the agility and innovation associated with the Adhocracy culture.

The diverse culture profile of the EMD also reflected a strategic alignment with the multifaceted needs of its patient population and operational demands. The presence of a Market culture within the EMD was advantageous as it provided the impetus for continuous improvement and responsiveness to market trends. The Adhocracy culture further enhanced this environment by promoting experimentation and risk-taking, enabling the EMD to develop novel approaches to patient care and operational challenges, which is crucial for rapidly evolving situations such as epidemic outbreaks. While the Hierarchy culture helps in bringing stability and structure to the organization, its lower scores from employees (22.62 points) aligned with the unique context of emergency medicine. While guidelines and hierarchical structures can provide a sense of order and consistency, flexibility is essential for accommodating the diverse and often unpredictable circumstances encountered emergency care. This balanced approach allows the EMD to maintain a structured framework while remaining agile enough to address the individual needs of each patient effectively.

### 4.2. Examination of Hypotheses

**Hypothesis** **1** **(H_1_).** *There will be no significant difference in cultural preferences among the employees in the EMD.*

**Interpretation**: A Kruskal–Wallis rank sum test was conducted to test this hypothesis, and the obtained *p*-value was found to be statistically significant (*p* < 0.05). Thus, we rejected the null hypothesis (H_1_) and confirmed that there was a significant difference in culture preferences among the EMD employees. Additionally, a Dunn test, which serves as the equivalent of a post hoc test in non-parametric tests, was performed to examine pairwise significant differences in culture preferences. The results revealed statistically significant differences among the Adhocracy–Clan, Adhocracy–Hierarchy, Clan–Hierarchy, Adhocracy–Market, Clan–Market, and Hierarchy–Market culture pairs with *p* < 0.005. This test confirmed the presence of a culture mix in the EMD, statistically.

**Hypothesis** **2** **(H_2_).** *There is an association between organizational culture and profession.*

**Interpretation:** A chi-square test was conducted to check the association between organizational culture and profession. The results did not yield a significant *p*-value, implying that profession does not influence cultural preferences in the EMD.

**Hypothesis** **3** **(H_3_).** *Employees perceiving a Clan culture will report higher job satisfaction and lower levels of burnout and stress compared to those in other cultural environments (H_3_).*

**Interpretation:** The Clan culture reported the highest overall job satisfaction (96.66%, *n* = 58) and lower levels of burnout [emotional exhaustion = 46.66%, *n* = 28 (sometimes); depersonalization = 53.33%, *n* = 32 (never); and a feeling of reduced personal accomplishment = 31.66%, *n* = 19 (rarely)]. A total of 48.33% (*n* = 29) of the employees perceiving a Clan culture felt low stress compared to other cultures, confirming our hypothesis.

### 4.3. Employee Behavior

This study discovered consistent behaviors among the employees regardless of their perceived culture type. Key behaviors included information sharing and collaboration, open communication, team support, inclusive decision making, constructive conflict resolution, commitment to patient-centric care, active problem solving, high adaptability in responding to emergent situations, continuous learning and knowledge seeking, respect for authority, and structured decision making. These findings suggested that the EMD had a strong organizational identity where core values and attributes shaped employee conduct across various cultures. These attributes and core values set the EMD apart from other organizations, leading employees to engage more in organizational citizenship behaviors [49]. The Clan culture’s emphasis on collaboration and communication permeated employee interactions even for those perceiving different cultures. In India, there is a strong emphasis on respecting elders and individuals in positions of authority. This social value likely permeated the organizational culture of the EMD, contributing to the observed behavior of respect for authority across all the cultural types represented within the organization. This finding of our study aligns with the findings of Yavuz Bolat et al., highlighting how social values play a vital role in shaping an organization’s culture [50]. Consistent structured decision making was a norm in the EMD where this study was conducted due to established standard operating procedures (SOPs). These SOPs provided a uniform framework for guiding employee behavior, facilitating structured decision making processes across the cultures within the EMD.

### 4.4. Job Satisfaction

A total of 87.80% of the employees expressed overall job satisfaction, with higher rates among those perceiving a Clan culture (96.66%). This contrasts with the findings of Kang Li et al., where 42.01% of healthcare personnels in the EMD expressed dissatisfaction [51]. While the study by Kang Li et al. does not specify whether job dissatisfaction arises from a toxic organizational culture, one possible explanation for the high rates of satisfaction in our study can be attributed to the fact that the majority of the employees (73.17%, *n* = 60) perceived themselves to be a part of a Clan culture, and this type of culture is associated with higher job satisfaction. A total of 98.78% of the employees found their work meaningful, which was a strong motivator for engagement and performance. In our study, high satisfaction with work alignment to personal values (98.33%, *n* = 59) was found among the employees perceiving a Clan culture, which fostered a supportive environment where individuals could express their unique strengths and preferences, leading to the customization of roles based on each employee’s skills and interests. This was evidenced by the lack of agreement among Clan culture employees regarding the emphasis on role clarity. A lower alignment satisfaction in other cultures indicated potential for improvement through higher involvement and autonomy. While recognition satisfaction was high, at 93.90%, it was lower for the Hierarchy culture. One potential explanation for this phenomenon could be a predisposition towards formal recognition ceremonies or structured programs. While these formalities can be meaningful for some employees, they may not resonate with everyone, especially those who prefer more informal or spontaneous forms of recognition. This highlights the need for better mechanisms to acknowledge contributions within structured settings.

### 4.5. Burnout

Emotional exhaustion (53.66%, *n* = 44) occurred sometimes, and depersonalization symptoms never (47.56%, *n* = 39) or rarely occurred (32.93%, *n* = 27) across cultures, while reduced personal accomplishment occurred sometimes (28.05%, *n* = 23). This indicates that employees are dedicated but may occasionally doubt their competence. The employees in a Market culture reported higher depersonalization (42.85%, *n* = 3), potentially due to its competitive nature. The overall burnout risk seems moderate but may rise if exacerbating factors like extreme patient volumes or inadequate staffing occur. Preventative strategies like workload management, peer support systems and communication of impact may therefore be beneficial.

### 4.6. Stress

While high workload and patient volumes emerged as the primary stressors for the employees perceiving a Clan culture (61.67%, *n* = 37), these employees reported lower overall stress levels. These lower stress levels can be attributed to the collaborative and team spirit of a Clan culture, in which employees rely on their collaborative instincts to support one another and share responsibilities, thereby reducing their stress levels. Their preference for seeking social support from colleagues or friends to reduce stress (63.33%, *n* = 38) further supports this theory of a resilience-conferring effect of Clan culture.

In contrast, Hierarchy culture employees (nurses) highlighted a lack of flexibility in decision making as a major source of stress (55.56%, *n* = 5). This indicates limiting autonomy in rigid structures. This observation is reinforced by the fact that nurses in India lack the same level of autonomy as physicians in clinical settings.

Market culture employees reported the highest stress levels (57.15%, *n* = 4), potentially resulting from constant pressure to achieve goals rapidly. While the dominant Clan culture promotes effective stress management, additional interventions to build resilience across cultures may be beneficial. Reducing stress can lead to higher productivity levels [52].

### 4.7. Coping Strategies

The employees perceiving a Clan culture favored seeking social support from colleagues or friends as their top strategy for coping with stress (63.33%, *n* = 38). This aligns with the collaborative and people-oriented nature of the Clan culture, where employees feel a strong sense of belonging and support, making it natural for them to leverage relationships to help manage stress. Time management (36.67%, *n* = 22), mindfulness techniques (46.67%, *n* = 28), and exercise (30%, *n* = 18) were also among the opted choices, reflecting a range of healthy coping strategies.

In the Adhocracy culture, collaborative problem solving, seeking support, and professional development ranked highest (66.67%, *n* = 4). The dynamic Adhocracy culture values risk-taking and innovation. Hence, the approach of employees to make use of social and individual strategies to deal with the stress is sensible, as working together with colleagues to solve problems can provide multiple perspectives and ideas, making it easier to find effective solutions to stressful situations. Additionally, investing in professional development enables employees to acquire skills and knowledge that enhance their ability to cope with stress and navigate challenging circumstances more effectively.

In the Market culture, seeking social support from colleagues or friends ranked highest (77.78%, *n* = 7). Seeking social support indicates that the employees in a Market-oriented cultures recognize the value of turning to others for emotional support, advice, and encouragement during stressful times. A total of 28.5% of the employees (*n* = 2) chose professional development as a method to reduce stress, aligning with the culture’s results-driven mentality.

In the Hierarchy culture, seeking guidance and clarification from superiors and seeking social support from colleagues and friends ranked highest, with 77.78% (*n* = 7) and 66.67% (*n* = 6), respectively. This pattern may stem from the perception that seeking guidance from superiors is essential for maintaining order and following established protocols. Additionally, the preference for seeking social support highlights that, despite the hierarchical structure, building strong relationships with colleagues and friends is still valued.

While social support emerged as a key coping mechanism in the workplace, irrespective of the organizational context, there were some clear cultural differences in the preferred coping strategies. Employees tend to leverage strategies well-aligned with the existing cultural values and norms. Organizations should promote culture-specific wellness initiatives, targeting the most favored strategies within each culture.

### 4.8. Study Limitations

This study had a few limitations, which are outlined below, along with corresponding future recommendations:

1. This study included employees from a single EMD. This single-setting study made it difficult to generalize the findings more broadly. Expanding this study across multiple hospitals could improve generalizability.
2. The specific breakdown of the sample (e.g., 8 physicians, 66 nurses, and 8 EMTs) resulted in uneven subgroups for comparison. Culture-specific insights were only available for nurses. Having a more balanced representation across roles could enable more robust role-based comparisons.
3. As this study collected data at one point in time, it provided only a snapshot compared to the longitudinal tracking of cultural patterns, employee experiences, and well-being over time. Repeated cultural examinations could elucidate cultural evolution.
4. This study exclusively utilized quantitative metrics around employee perceptions, frequencies, and agreements. Adding a qualitative component like interviews could provide richer, contextualized insights into individuals’ cultural experiences.

### 4.9. Recommendations for Potential Improvements to Enhance Employee Well-Being and Overall Performance in the EMD

1. Establish an on-site professional counseling service consisting of licensed therapists and psychologists that can provide emergency medicine department staff with easily accessible mental health support and evidence-based stress management training on a regular basis. This will help in promoting positive coping skills and resilience.
2. Establish a structured mentorship and coaching program to provide guidance and support for less experienced employees. This can help mitigate stress, impart coping skills, and facilitate career advancement.
3. Provide professional development stipends and allot time towards continuing education to nurture growth and career progression. This can motivate staff to pursue further education and training, fostering personal and professional development.
4. Recognize employee milestones through department-level awards, ceremonies, and newsletters to foster inclusivity, praise contributions, and boost morale. This will help in creating a culture of inclusivity and appreciation within the department.
5. Establish a peer support program that trains veteran staff as mentors to lend social support to struggling colleagues and impart effective coping techniques. Veteran staff, through their experience, can offer guidance, advice, and a listening ear to struggling colleagues, helping them navigate work-related stressors and build resilience.

## Figures and Tables

**Table 1:** Culture profile of EMD.

Culture	Scores
Clan	30.81
Market	23.41
Adhocracy	23.15
Hierarchy	22.62
**Total**	**100.00**

**Table 2:** Distribution of organizational culture.

Culture	No. of Participants	% of Total
Clan	60	73.17%
Adhocracy	6	7.31%
Market	7	8.54%
Hierarchy	9	10.98%

**Table 3:** Profession-wise distribution of organizational culture.

Culture	Profession	No. of Employees	% of Total (*n* = 82)
Clan	Physician	8	100%
	Nurse	44	66.66%
	EMT	8	100%
Adhocracy	Nurse	6	9.09%
Market	Nurse	7	10.61%
Hierarchy	Nurse	9	13.64%

**Table 4:** Consistent behaviors exhibited by the employees in EMD.

SN	Exhibited Behavior	Average Rating
1	Information sharing and collaboration	4.73
2	Open communication and active listening	4.45
3	Team support and adaptability	4.76
4	Inclusive decision making	4.23
5	Constructive conflict resolution	4.32
6	Active problem solving	4.17
7	High adaptability in responding to emergent situations	4.16
8	Continuous learning and knowledge seeking	4.10
9	Outcome prioritization	4.38
10	Adaptability to current market trends	4.04
11	Commitment to patient-centric care	4.37
12	Respect for authority	4.48
13	Structured decision making	4.37

**Table 5:** Job satisfaction agreement by organizational culture type.

SN	Organizational Culture	Average Rating				
		Meaning and Fulfillment in Work	Recognition and Value for Contributions	Satisfaction with Work Environment and Culture	Alignment of Work with Personal Values and Goals	Overall Job Satisfaction
1	Clan culture	60 (100%)	57 (95%)	59 (98.33%)	59 (98.33%)	58 (96.66%)
2	Adhocracy culture	6 (100%)	6 (100%)	5 (83.33%)	5 (83.33%)	5 (83.33%)
4	Market culture	6 (85.71%)	7 (100%)	5 (71.42%)	5 (71.42%)	4 (57.14%)
4	Hierarchy culture	9 (100%)	7 (77.77%)	6 (66.66%)	6 (66.66%)	5 (55.55%)

**Table 6:** Frequency of emotional exhaustion by organizational culture perception.

SN	Organizational Culture	Emotional Exhaustion				
		Never	Rarely	Sometimes	Often	Very Often
1	Clan culture	7 (11.66%)	19 (31.66%)	28 (46.66%)	6 (10%)	0 (0%)
2	Adhocracy culture	0 (0%)	1 (16.66%)	5 (83.33%)	0 (0%)	0 (0%)
3	Market culture	1 (14.28%)	0 (0%)	5 (71.42%)	1 (14.28%)	0 (0%)
4	Hierarchy culture	1 (11.11%)	2 (22.22%)	6 (66.66%)	0 (0%)	0 (0%)

**Table 7:** Frequency of depersonalization by organizational culture perception.

SN	Organizational Culture	Depersonalization				
		Never	Rarely	Sometimes	Often	Very Often
1	Clan culture	32 (53.33%)	20 (33.33%)	5 (8.33%)	2 (3.33%)	1 (1.66%)
2	Adhocracy culture	3 (50%)	1 (16.66%)	2 (33.33%)	0 (0%)	0 (0%)
3	Market culture	0 (0%)	2 (28.57%)	2 (28.57%)	3 (42.85%)	0 (0%)
4	Hierarchy culture	4 (44.44%)	4 (44.44%)	1 (11.11%)	0 (0%)	0 (0%)

**Table 8:** Frequency of reduced personal accomplishment by organizational culture perception.

SN	Organizational Culture	Reduced Personal Accomplishment				
		Never	Rarely	Sometimes	Often	Very Often
1	Clan culture	17 (28.33%)	19 (31.66%)	15 (25%)	8 (13.33%)	1 (1.66%)
2	Adhocracy culture	1 (16.66%)	2 (33.33%)	2 (33.33%)	1 (16.66%)	0 (0%)
3	Market culture	1 (14.28%)	3 (42.85%)	2 (28.57%)	0 (0%)	1 (14.28%)
4	Hierarchy culture	3 (33.33%)	1 (11.11%)	4 (44.44%)	1 (11.11%)	0 (0%)

**Table 9:** Overall frequency of burnout components in the EMD.

SN	Burnout Criteria	Frequency	No. of Employees	Percentage of Employees
1	Emotional exhaustion	Never	9	10.98%
		Rarely	22	26.83%
		Sometimes	44	53.66%
		Often	7	8.53%
		Very often	0	0%
2	Depersonalization	Never	39	47.56%
		Rarely	27	32.93%
		Sometimes	10	12.20%
		Often	5	6.10%
		Very often	1	1.21%
3	Reduced personal accomplishment	Never	22	26.83%
		Rarely	25	30.49%
		Sometimes	23	28.05%
		Often	10	12.20%
		Very often	2	2.43%

**Table 10:** Employee perceptions of organizational culture and stress levels.

SN	Organizational Culture	No. of Employees				
		Very Low Stress	Low Stress	Moderate Stress	High Stress	Very High Stress
1	Clan culture	8 (13.33%)	29 (48.33%)	20 (33.33%)	3 (5%)	0 (0%)
2	Adhocracy culture	1 (16.66%)	2 (33.33%)	2 (33.33%)	1 (16.66%)	0 (0%)
3	Market culture	0 (0%)	1 (14.28%)	2 (28.57%)	4 (57.14%)	0 (0%)
4	Hierarchy culture	0 (0%)	1 (11.11%)	4 (44.44%)	4 (44.44%)	0 (0%)

## Data Availability

Data is available from the corresponding author on request.
